# Mutual wellbeing in an equine-assisted intervention: experimental evidence for the two-way hypothesis and the importance of interaction method

**DOI:** 10.1038/s41598-026-65168-2

**Published:** 2026-09-15

**Authors:** Claire J. M. Short, Rebecca M. Calisi

**Affiliations:** Department of Neurobiology, Physiology and Behavior, College of Biological Sciences, University of California, Davis, USA

**Keywords:** Neuroscience, Psychology, Psychology, Zoology

## Abstract

Rising rates of mental health disorders have increased demand for scalable, non-pharmacological interventions. Interactions with animals can improve emotional wellbeing across diverse populations, yet the mechanisms responsible for these benefits remain poorly understood. Most research focuses on human outcomes while treating the animal as a passive component of the interaction, despite human–animal interactions being inherently reciprocal. We therefore propose the Two-Way Hypothesis, which predicts that human behavior shapes animal behavior, which in turn contributes to variation in human emotional outcomes. We experimentally tested this hypothesis in 61 adolescents who participated in standardized interactions with miniature equines (horses and donkeys) under manipulated tactile conditions (petting or scratching). Emotional state was assessed before and after the interaction, and equine behavioral responses were recorded during the interaction. Scratching increased affiliative lip-wiggling behavior and reduced voluntary disengagement (walking away) compared with petting. In turn, observing lip wiggling predicted greater reductions in loneliness, whereas observing walking away predicted greater reductions in sadness. These findings provide experimental support for the Two-Way Hypothesis, demonstrating that human interaction style can influence animal behavior and that this behavioral variation corresponds to differences in human emotional outcomes. This framework provides a tractable basis for investigating the mechanisms underlying animal-assisted interventions.

**Supplementary Information:**

The online version contains supplementary material available at https://doi.org/10.1038/s41598-026-65168-2.

## Introduction

Human-animal interactions are widely used to support mental health in contexts ranging from clinical therapy to educational and community programs. These approaches, often described as animal-assisted interventions (AAIs) and more recently as animal-assisted services (AAS)^1^, intentionally incorporate animals into structured activities designed to improve human wellbeing. They have been associated with improvements in mood, emotional regulation, and symptoms of depression, anxiety, and post-traumatic stress^2–8^. These benefits have been reported across diverse populations, from children to adults, suggesting that AAIs may represent a broadly applicable approach to supporting emotional health^3,9–11^.

However, findings across studies vary considerably, pointing to an incomplete understanding of the mechanisms through which these interactions produce their effects^2,12–14^. One reason for this gap is that studies of human-animal interaction typically evaluate human outcomes without accounting for variation in the animal’s behavior during the interaction. Animal welfare is generally treated as an ethical prerequisite or control variable rather than a potential driver of variation in human responses, thereby framing animals as passive components of the interaction rather than responsive participants. Recent conceptual work has begun to challenge this view, emphasizing that animals in these contexts are not passive tools but active participants whose behavior can shape the quality and outcomes of interactions^1,15^. If so, then variation in animal behavior is not peripheral to the interaction but may be one pathway through which human outcomes arise. This perspective shifts attention from mere exposure to animals toward the dynamics of the interaction itself, positioning those dynamics as central to understanding how human-animal interactions produce psychological effects.

**Fig. 1:**
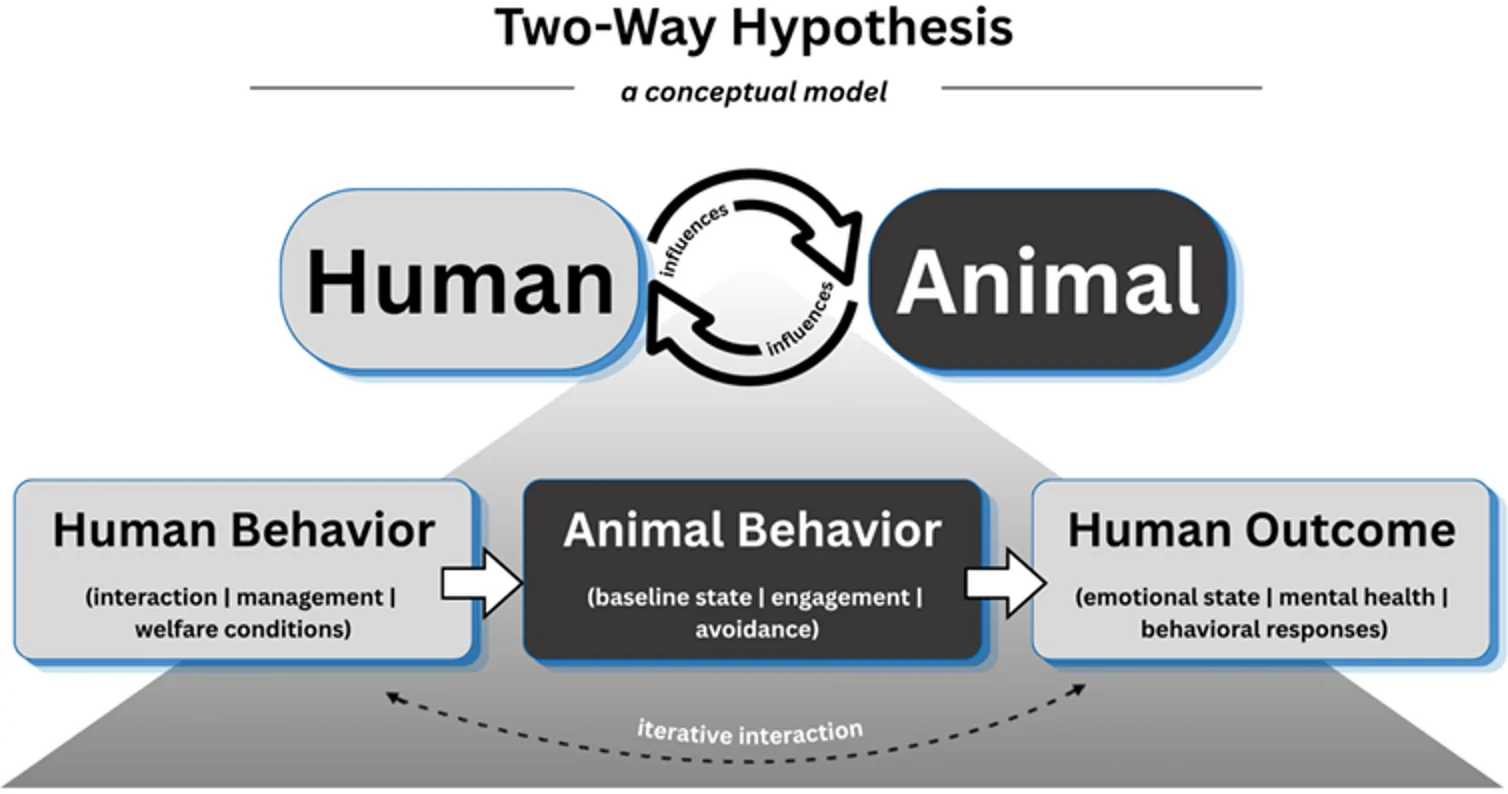
Conceptual model of the Two-Way Hypothesis in human–animal interaction. Human-animal interactions are depicted as a reciprocal system in which both agents influence one another, while also forming a directional behavioral pathway from human behavior to human outcomes via animal behavior. Human actions, including interaction style, management, and welfare conditions, shape animal behavioral responses, which in turn contribute to variation in human emotional state, mental health, and behavior. The dashed arc indicates iterative feedback over time, whereby outcomes from one interaction may influence subsequent human behavior.

To account for this dynamic, we have developed a Two-Way Hypothesis of human-animal interaction (Fig. 1). The hypothesis proposes a pathway in which human behavior and interaction design shape the animal’s experience, and that experience, in turn, contributes to variation in human outcomes. Within this framework, variation in animal behavioral responses, such as engagement, avoidance, or affiliative behavior, may explain variability in human emotional responses.

Equines, including the domestic horse (*Equus ferus caballus*) and donkey (*Equus asinus*) are among the most commonly used animals in AAIs^16–20^ and are highly sensitive to human behavioral cues^21,22^. The present study used miniature horses and miniature donkeys, as their smaller size facilitates close, ground-based interactions, while their sensitivity to human behavior makes them a tractable system for experimentally testing whether variation in human behavior shapes animal responses and, in turn, human outcomes. Although miniature horses are commonly represented by breeds such as the American Miniature Horse and miniature donkeys by the Miniature Mediterranean Donkey, the breed ancestry of the study animals was unknown. Equine-assisted interventions have been associated with improvements in psychological wellbeing^3,4,9,11,20^, yet it remains unclear whether these benefits depend, in part, on how humans interact with the animals.

The present study provides an experimental test of the Two-Way Hypothesis using brief interactions between adolescents and miniature equines. Adolescence represents a critical developmental period for emotional wellbeing^23,24^ during which early support may help prevent the development or progression of mental health challenges^25^. Miniature equines may be particularly well suited for supporting emotional regulation in adolescents given their smaller size and suitability for close, ground-based interaction, yet their use in AAIs remains relatively understudied.

To test whether variation in equine engagement is associated with variation in human emotional outcomes, we experimentally manipulated tactile interaction style during a structured three-minute interaction between adolescents and miniature equines. Participants were randomly assigned to either a petting condition (gentle stroking along the equine’s back) or a scratching condition (firmer contact along the neck).

Prior work has demonstrated that equines exhibit differential behavioral and physiological responses to tactile stimulation, including affiliative responses to contact that mimics allogrooming between equines and avoidance of less preferred forms of handling^22,26–29^. Based on this work, as well as preliminary observations from the present study context, we predicted that equines in the scratching condition would be more likely to display affiliative behavior and less likely to disengage from the interaction than those in the petting condition. We further hypothesized that greater equine engagement would correspond to larger improvements in human emotional state.

By experimentally manipulating human behavior and measuring both animal responses and human outcomes, this study provides a framework for understanding the mechanisms underlying AAIs. By identifying animal behavior as a measurable intermediate process linking human action to human outcomes, these findings offer a foundation for developing more effective, scalable, and mechanistically informed intervention strategies.

## Methods

### Participants and study context

Sixty-one adolescents participated in the study during a week-long animal behavior summer program hosted by the nonprofit animal rescue Skyheart Sanctuary at Pine Trails Ranch in Davis, California. Participants were enrolled in one of several camp sessions held during summer 2025. All participants provided assent, and written parental consent was obtained prior to participation. Demographic information, including age, gender identity, and ethnicity, was collected for descriptive purposes and to evaluate potential confounding variables.

All equine interaction sessions were scheduled to standardize both experience and context. Sessions took place on the first day of each camp session, before participants had substantial prior exposure to the animals, and were conducted between 10:00 and 11:00 AM to reduce potential time-of-day effects on mood and heart rate. Each participant completed pre- and post-interaction assessments of emotional state, and human participant heart rate was recorded at both time points.

### Study design and procedure

This study was designed to test the Two-Way Hypothesis by evaluating whether experimentally induced differences in human interaction would alter equine behavioral responses and whether those responses would, in turn, correspond to variation in adolescent emotional outcomes.

To enable participants to recognize equine behavioral cues, each session began with a brief standardized training. Before the interaction, participants received the same ~ 10-minute lesson introducing two target behaviors: upper-lip extension or twitching, hereafter referred to as “lip wiggling,” and moving away from the participant, hereafter referred to as “walking away.” Lip wiggling was presented as a potential indicator of positive emotional states in equines^27^, and walking away was interpreted as an avoidance or disengagement behavior^29^. Instruction included photographs, a video of the lip-wiggling behavior, and brief verbal descriptions, emphasizing observation of the animal’s behavior during the interaction rather than subjective interpretation. Participants then underwent a brief instructor-led quiz to confirm understanding.

Following this standardized instruction, baseline measures were collected. Participants completed a pre-interaction survey assessing emotional state (Supplementary Appendix A), and baseline heart rate was recorded. Participants were then randomly assigned to one of two tactile conditions: petting or scratching. Tactile interaction was standardized using condition-specific instructions. In the petting condition, participants were instructed to gently stroke the equine’s back using a smooth, continuous motion. In the scratching condition, participants were instructed to apply firmer, localized contact to the neck using small, repetitive movements of the fingers, mimicking allogrooming^26^. Participants were encouraged to maintain engagement with the animal while adhering to their assigned interaction style. To preserve the manipulation, participants were told that these instructions standardized the interaction and were not informed that multiple conditions existed.

Participants then began the interaction phase. They entered the enclosure in small groups of two to four and were allowed to interact freely with one or more miniature equines for a duration of three minutes. The equine group consisted of four animals: two miniature donkeys and two miniature horses, each species represented by a mother-offspring pair. The adult females were rescue animals of unknown age, whereas the male offspring were approximately three years old at the time of the study. All four equines had cohabited within the same enclosure at the sanctuary for more than two years before the study and were well habituated both to one another and to routine interactions with human visitors. The adolescents were advised not to interact with one individual, who was more reserved and less likely to seek interaction. This was done to respect her comfort and voluntary level of participation, consistent with the study’s emphasis on animals as active participants.

The experiment was structured to permit natural variation in equine behavior. Interactions took place in the animals’ outdoor home enclosure (20 × 40 ft), where the equines remained unrestrained throughout the procedure. Participants were allowed to remain with a single equine or move among animals, while equines were free to approach, maintain engagement, or disengage by moving away. This design enabled equine behavioral responses to vary naturally within the constraints of the assigned tactile manipulation, preserving the animals’ capacity for voluntary engagement and disengagement. Speaking to the animals was permitted; however, conversation among participants was discouraged to minimize potential social influences on emotional responses while maintaining a naturalistic interaction context. All sessions were conducted by the same female experimenter, who had worked extensively with the study equines for more than two years before the study, minimizing potential variation associated with experimenter identity and familiarity^30,31^. The experimenter intervened only as needed to ensure safety and adherence to the assigned tactile condition.

Immediately following the interaction, outcome measures were collected. Participants exited the enclosure and completed a post-interaction survey (Supplementary Appendix B), and heart rate was recorded at a second time point. In addition to emotional state measures, the post-interaction survey included items assessing whether specific equine behaviors, including lip wiggling and walking away, were observed. This design captured both participants’ emotional responses and their perception of equine behavior within the same interaction.

### Human emotional and physiological measures

Emotional state was assessed immediately before and after the interaction using five self-report Likert-type items. Participants were asked to rate the extent to which they felt nervous/anxious, sad, excited, lonely, and calm on a scale from 0 (“not at all”) to 7 (“extremely”). Each item was analyzed separately rather than combined into a composite index to preserve distinctions among emotional dimensions that could plausibly respond differently to the interaction^32–34^.

Heart rate was measured immediately before and after the interaction using a fingertip pulse oximeter (Zacurate 500BL, Zacurate, USA), which provides non-invasive estimates of pulse rate via photoplethysmography. Values below 40 beats per minute or above 180 beats per minute were excluded as implausible, consistent with probable device malfunction or recording error. Blood oxygen saturation readings were used only to verify device functionality and were not included in further analyses.

### Equine behavioral measures

Participants reported whether they observed lip wiggling and whether any equine walked away during the interaction. These behaviors were selected because they are discrete and readily identifiable, reducing ambiguity in participant reporting. Because participants were free to interact with one or more equines, these measures reflect equine behavior as perceived by the participant within the interaction, rather than behavior linked to a specific individual. Accordingly, the measures capture equine behavior as perceived by the participant, the dimension most directly relevant to shaping human emotional responses^35–38^.

These behaviors served as indicators of equine engagement within the interaction. Lip wiggling, defined here as repetitive movements of the upper lip during tactile contact, has been described in horses during preferred grooming interactions and interpreted as a marker of positive affective states^27^. Avoidance was indexed by voluntary disengagement, defined as the equine walking away from the participant during the interaction^29^. Together, these cues provided observable proxies for the animal’s apparent experience during the interaction.

Immediately following the interaction, participants exited the enclosure and completed the post-interaction survey, which included binary questions asking whether they had observed each target equine behavior (lip wiggling and walking away) during the interaction. Participants did not record behaviors while interacting with the animals, allowing the interaction to proceed uninterrupted. Participant reports of lip wiggling and walking away were therefore used as post-interaction indicators of perceived equine engagement and disengagement, respectively. Importantly, both tactile manipulations and resulting equine behaviors were designed to remain within welfare-positive interaction bounds. Behavioral variables were coded as binary indicators (1 = observed, 0 = not observed). To reduce misclassification, a value of 0 was assigned only when the participant reported not observing the behavior. When an individual could not confidently evaluate that animal’s behavior, the observation was treated as structurally missing rather than as a true absence.

### Statistical analyses

Analyses were conducted to address three primary questions: (1) whether the interaction improved emotional state overall, (2) whether the manipulation of human behavior influenced equine behavior, and (3) whether variation in equine behavior, in turn, was associated with variation in human outcomes.

To evaluate overall emotional change, linear mixed-effects models were used with time (pre vs. post) as a fixed effect with participant as a random intercept. Camp week was included as an additional random effect to account for potential cohort-level variation. All tests were two-tailed with α = 0.05, as directional effects were not assumed for all individual emotional outcomes despite a priori hypotheses regarding overall patterns of change. Emotional outcomes were analyzed separately, as they represent conceptually distinct dimensions of emotional experience rather than repeated measurements of a single underlying construct^32–34^, consistent with multidimensional models of affect^34^. Accordingly, corrections for multiple comparisons were not applied. Such adjustments are most appropriate when multiple tests evaluate the same underlying construct, whereas the present analyses examine prespecified, theory-driven outcomes that are not interchangeable^39,40^. In this context, strict correction would increase the risk of Type II error and reduce power to detect meaningful effects^39–41^. Results were therefore interpreted based on the consistency and direction of effects across outcomes, rather than reliance on isolated p-values^42^.

To test whether the tactile manipulation altered equine behavior, generalized linear models with a binomial error structure were used to evaluate whether the probability of observing lip wiggling or walking away differed between conditions. Models were estimated at the level of the individual participant with cluster-robust standard errors by camp week to account for non-independence within sessions. Effect sizes are reported as odds ratios (ORs) with corresponding p-values.

To test the Two-Way Hypothesis, baseline-adjusted linear models were used to evaluate whether equine behaviors, influenced by experimentally induced differences in human interaction, were associated with variation in post-interaction emotional outcomes. For each emotional variable, post-interaction scores were modeled as a function of the corresponding pre-interaction score and observed equine behaviors. This approach evaluates whether variation in equine responses corresponds to variation in participant emotional state.

Rather than relying on formal mediation analysis, which would require strong assumptions about unmeasured confounding^43^ and sufficient power to detect indirect effects^44^, the study experimentally manipulated the upstream component of the proposed pathway. This approach provides mechanistic leverage by linking experimentally induced differences in human behavior to variation in animal responses and those responses, in turn, to human outcomes. Although causal inference for the downstream component (animal behavior → human outcome) remains limited, it represents an important direction for future work.

The use of a consistent group of equines reduced variability in the interaction environment across participants. Because the number of animals was small and interaction exposure varied across participants, analyses were conducted at the participant level and did not model species- or individual-level effects.

Analyses were conducted in Python 3.11 using pandas, numpy, scipy, and statsmodels. The de-identified dataset and analysis scripts are publicly available at the project GitHub repository: https://github.com/BeccaCalisi/equine-interaction-two-way-hypothesis.git.

### Ethics statement

All procedures involving human participants were approved by the University of California, Davis Institutional Review Board (IRB #2213104-1) and conducted in accordance with relevant guidelines and regulations. Written informed consent was obtained from parents or legal guardians, and assent was obtained from all participants. All procedures involving animals were approved by the University of California, Davis Institutional Animal Care and Use Committee (IACUC #24019) and conducted in accordance with relevant guidelines and regulations.

## Results

### Participant characteristics

The sample was diverse in gender and ethnicity. Participants identified as 67% female, 25% male, and 7% nonbinary or gender-fluid. Reported ethnic identities were 52% White, 16% Asian or Asian American, 13% Hispanic/Latino/a/x, 13% Black (multiracial), and 6% not reported. Eight adolescents were reported by parents to be neurodivergent (autism spectrum disorder, ADHD, or both).

Demographic variables did not differ by tactile condition (petting vs. scratching), camp week, or observed equine-engagement patterns (all *p* > 0.10). Including demographic covariates in preliminary models did not change any substantive results. Mean heart rate also did not differ by demographic or experimental factors (all *p* > 0.10). Accordingly, demographic variables and heart rate were not included in final models to preserve statistical power.

### Effects of the interaction on adolescent emotional state

**Fig. 2:**
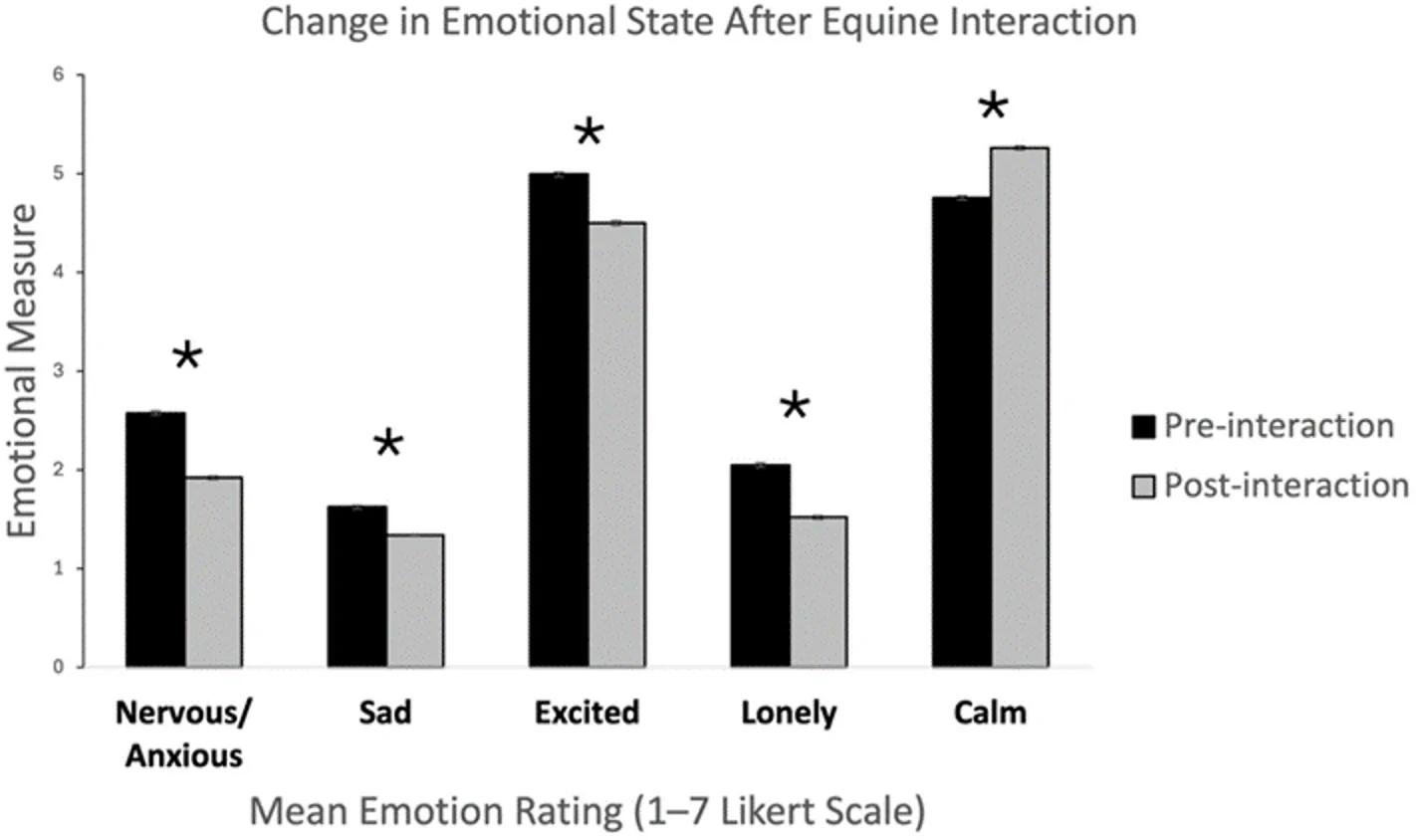
Changes in adolescent emotional state following a brief equine interaction. Mean self-reported emotion ratings before (pre-interaction) and after (post-interaction) a three-minute interaction with miniature equines. Ratings were measured on a 1–7 Likert scale for five emotional states: nervous/anxious, sad, excited, lonely, and calm. Bars represent mean scores across participants, with error bars indicating the standard error of the mean. Asterisks indicate statistically significant pre-post differences (*α* = 0.05).

A total of 61 participants provided complete pre- and post-interaction emotional ratings and were included in the analyses of emotional change. Consistent with the first analytic aim, emotional state shifted following the interaction. Across all five measures, participants reported significant shifts in emotional state after the three-minute equine interaction (Fig. 2).

Participants reported significantly lower levels of nervousness/anxiety after the interaction (pre: M = 2.57, SD = 1.43; post: M = 1.97, SD = 1.21; β = −0.607, SE = 0.134, *p* < 0.001). Sadness also decreased (pre: M = 1.62, SD = 1.10; post: M = 1.33, SD = 0.65; β = −0.295, SE = 0.114, *p* = 0.010), as did loneliness (pre: M = 2.05, SD = 1.48; post: M = 1.59, SD = 1.13; β = −0.459, SE = 0.110, *p* < 0.001). Participants also reported a reduction in excitement (pre: M = 4.99, SD = 1.31; post: M = 4.52, SD = 1.51; β = −0.467, SE = 0.145, *p* = 0.001), while calmness increased (pre: M = 4.75, SD = 1.22; post: M = 5.39, SD = 1.11; β = 0.639, SE = 0.129, *p* < 0.001).

**Fig. 3:**
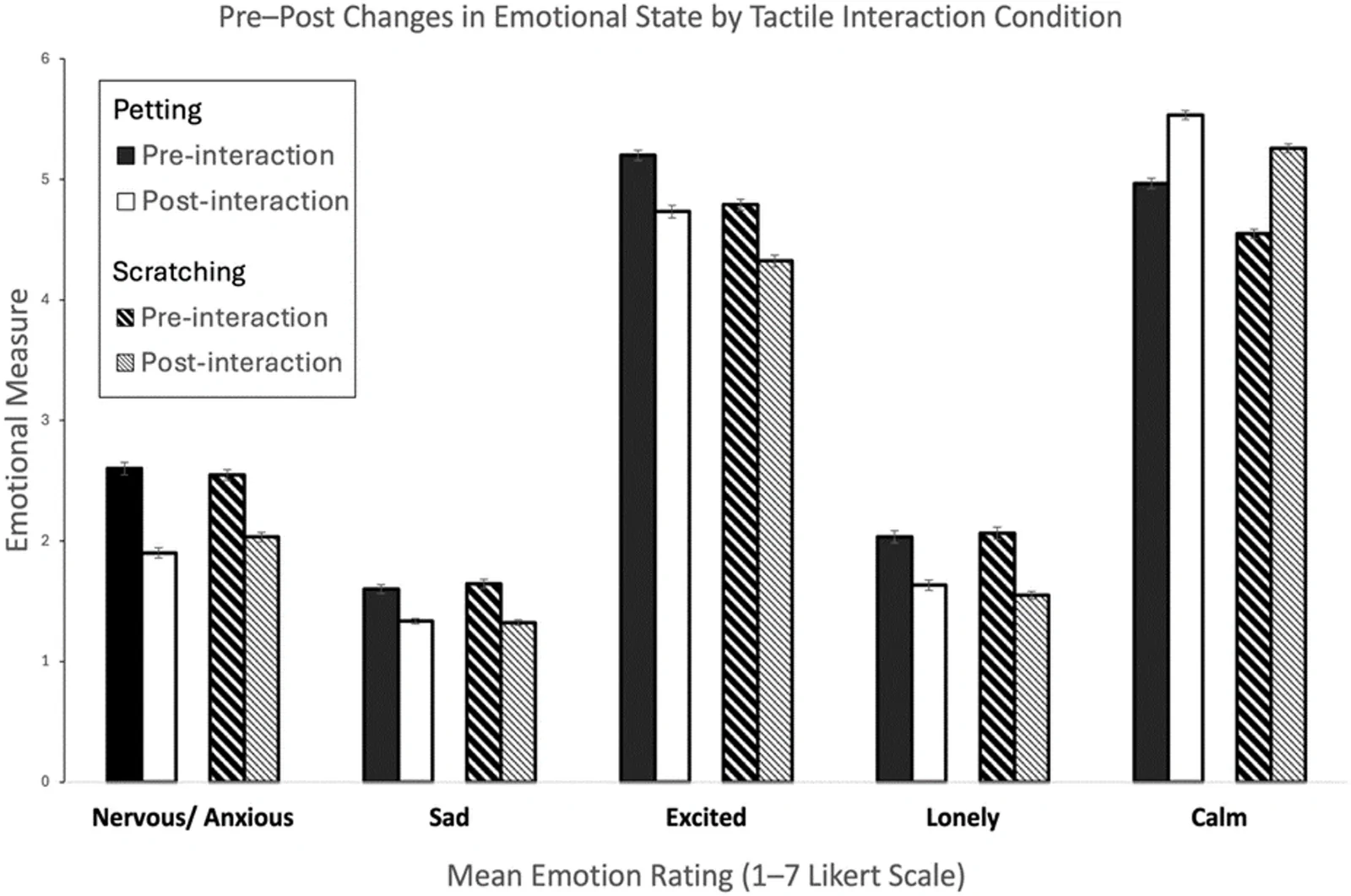
Pre-post emotional ratings by tactile interaction condition. Mean emotion ratings before and after the interaction for participants in the petting and scratching conditions. Both groups showed similar directional changes across all emotional measures. Bars represent mean scores across participants, with error bars indicating the standard error of the mean. Statistical tests evaluating predictors of emotional outcomes are reported in the main analyses and summarized in Fig. 4.

Collectively, these results demonstrate that even a brief equine interaction was associated with measurable improvements in multiple dimensions of emotional state. These pre-post shifts were observed in both tactile conditions, with participants in both petting and scratching groups showing changes in the same direction (Fig. 3). This overall improvement provided the context for testing whether variation in equine behavior, arising in part from differences in human interaction, explained differences in the magnitude of these changes.

### Effects of tactile manipulation on equine behavior

Behavioral analyses were conducted on the subset of adolescents who provided complete responses to the equine behavioral questions (*n* = 51). Surveys in which one or both behavioral items were left unanswered were treated as structurally missing for those variables and excluded from the corresponding analyses. Consistent with the experimental design, tactile condition (manipulating human behavior) influenced equine behavioral responses.

**Fig. 4:**
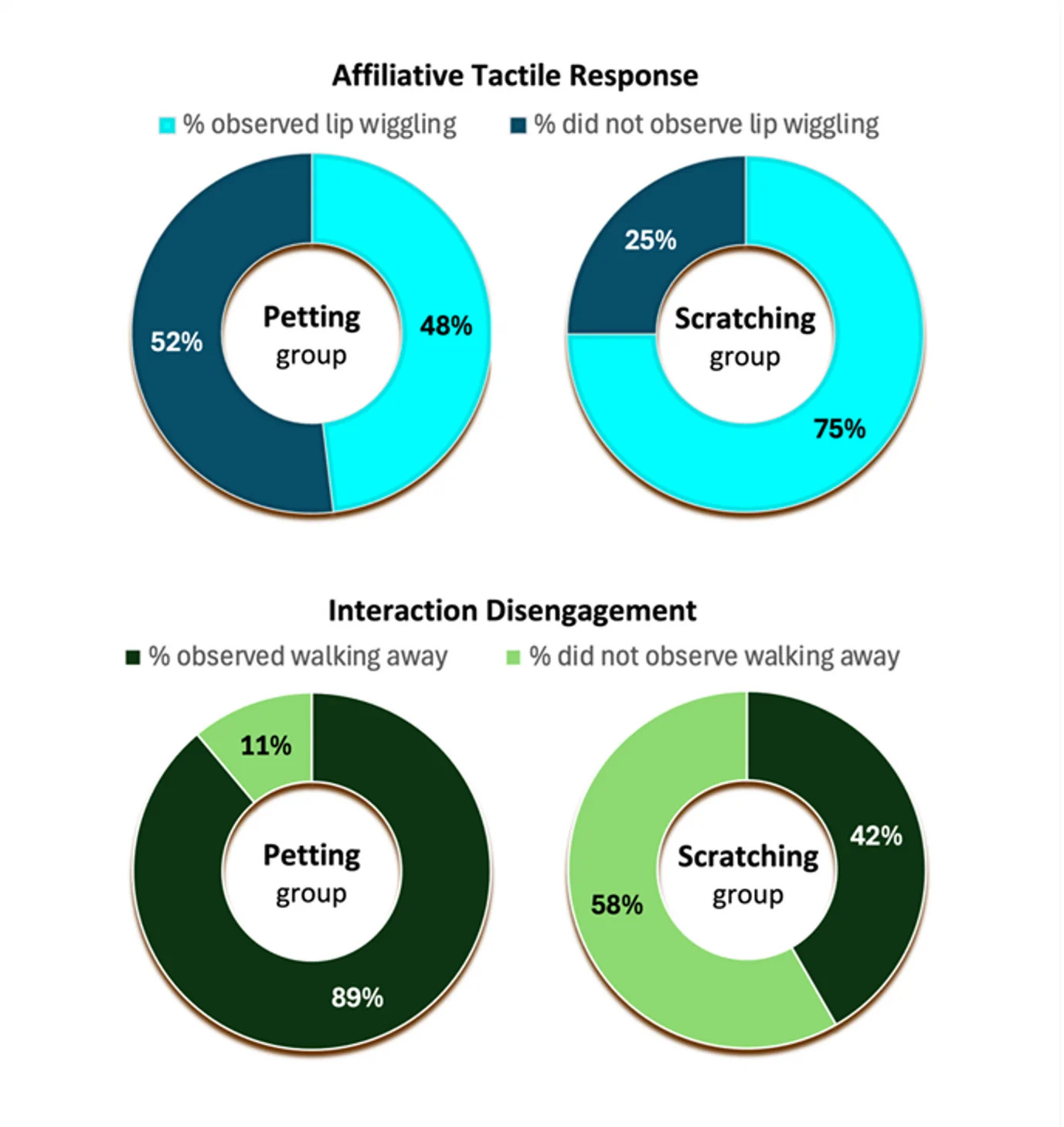
Effects of tactile interaction style on equine affiliative and disengagement behaviors. The proportion of participants reporting equine affiliative tactile responses (lip wiggling; top panel) and interaction disengagement (walking away; bottom panel) during a three-minute interaction with miniature equines. Donut charts show the percentage of participants who observed each behavior in the petting and scratching conditions. Percentages reflect the proportion of participants within each tactile condition who reported observing the behavior.

Reports of equine disengagement (walking away from the interaction) were significantly less frequent in the scratching condition than in the petting condition (41.7% vs. 88.9%; OR = 0.089, 95% CI [0.04, 0.20], *p* < 0.001; Fig. 4). Individuals in the scratching condition were also more likely to report observing lip wiggling than those in the petting condition (75.0% vs. 48.1%; odds ratio [OR] = 3.23, 95% CI [0.66, 15.76]). This sizable difference did not reach statistical significance in the clustered model (*p* = 0.073), although the estimated effect was in the predicted direction and associated with a large odds ratio. These results demonstrate that the manipulation of human behavior generated measurable variation in equine engagement, establishing an experimental basis for evaluating whether these differences correspond to variation in human outcomes.

### Relationship between equine behavior and human emotional outcomes

Baseline-adjusted models evaluated whether the magnitude of change in emotional outcomes varied as a function of equine behavioral responses. Because emotional state improved across participants regardless of condition (Fig. 3), these analyses test whether the degree of improvement differed as a function of equine behavior. Pre-interaction emotional scores were included as covariates in all models, ensuring that observed differences reflect variation in change rather than differences in baseline levels. Against this shared backdrop of improvement, specific equine behaviors covaried with differences in particular emotional outcomes.

**Fig. 5:**
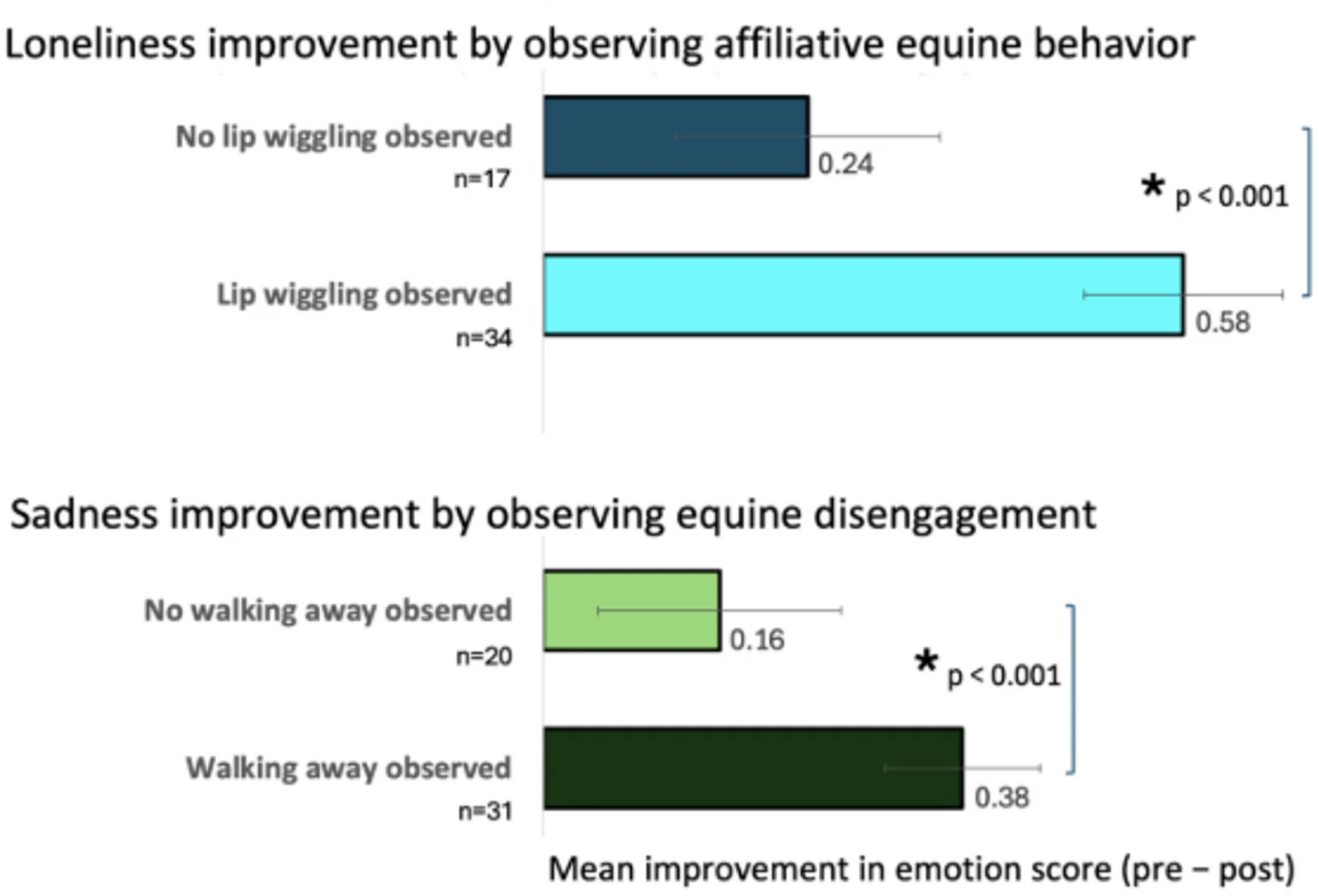
Changes in adolescent emotional state associated with observed equine behavioral responses during the interaction. Mean improvement in sadness and loneliness scores (pre-post) among participants who did or did not observe specific equine behaviors during the three-minute interaction. Top panel shows changes in sadness as a function of whether the equine voluntarily disengaged by walking away. Bottom panel shows changes in loneliness as a function of whether participants observed affiliative lip-wiggling behavior. Bars represent mean improvement in emotion scores (pre-interaction minus post-interaction), such that higher values indicate greater reductions in the reported emotion. Error bars represent standard errors of the mean. Sample sizes for each group are indicated. Asterisks denote statistically significant associations from baseline-adjusted regression models predicting post-interaction emotional scores while controlling for baseline values (*p* < 0.001).

Observing lip wiggling during the interaction was associated with significantly lower post-interaction loneliness after controlling for baseline (β = −0.215, SE = 0.059, 95% CI [− 0.33, − 0.10], *p* < 0.001; Fig. 5). Adolescents who observed lip wiggling reported loneliness scores that were, on average, 0.215 points lower than those who did not, holding baseline constant. This pattern indicates that affiliative equine behavior corresponded to greater reductions in loneliness. In contrast, observing the equine walk away was associated with lower post-interaction sadness (β = −0.115, SE = 0.028, 95% CI [− 0.17, − 0.06], *p* < 0.001; Fig. 5), reflecting greater reductions in sadness among participants who observed disengagement, within an overall improving sample.

For the remaining emotional outcomes, equine behavioral responses were not significantly associated with post-interaction emotional scores after controlling for baseline values. Lip wiggling did not predict nervousness/anxiety (β = −0.101, 95% CI [− 0.40, 0.20], *p* = 0.511), sadness (β = −0.206, 95% CI [− 0.57, 0.16], *p* = 0.273), excitement (β = −0.056, 95% CI [− 0.73, 0.62], *p* = 0.872), or calmness (β = 0.149, 95% CI [− 0.22, 0.52], *p* = 0.431). Similarly, walking away was not associated with nervousness/anxiety (β = −0.287, 95% CI [− 0.84, 0.27], *p* = 0.313), excitement (β = −0.086, 95% CI [− 0.44, 0.27], *p* = 0.632), loneliness (β = −0.070, 95% CI [− 0.42, 0.28], *p* = 0.695), or calmness (β = 0.155, 95% CI [− 0.27, 0.58], *p* = 0.478).

Thus, while emotional improvements were observed in both participant groups, the degree of improvement for specific emotions was associated with differences in equine behavioral responses. The variation in those responses arose at least in part from how participants engaged with the animals. These patterns are consistent with a proposed causal pathway in which human behavior shapes animal responses which, in turn, relate to human outcomes.

## Discussion

The present study introduces and provides support for a Two-Way Hypothesis of human-animal interaction, which conceptualizes these interactions as bidirectional processes. By experimentally manipulating human interaction, we found that interaction style influenced equine behavior, and that variation in these behavioral responses corresponded to differences in the magnitude of human emotional benefits.

Human-animal interactions are inherently multisensory and context-dependent, making it difficult to attribute effects to any single component. The present study therefore focused on how variation in these emotional responses arises within the interaction itself. As the goal was to examine variation within the interaction rather than to test the effects of animal presence versus absence, a non-animal control condition was not included. Instead, the study leveraged experimental control over human interaction to generate systematic variation in interaction dynamics, while using a pre-post design in which participants served as their own controls.

Within this framework, the observed improvements in adolescent emotional state are consistent with a growing body of evidence that interactions with animals, including brief and structured encounters, can produce measurable short-term effects^6,7,45,46^. After only three minutes of contact with miniature equines, adolescents reported lower nervousness, sadness, and loneliness, along with increased calmness. Participants also reported lower excitement, a pattern that likely reflects a reduction in anticipatory arousal rather than a loss of positive affect^47–50^, as many appeared visibly eager before entering the enclosure. Because human-animal interactions are most often studied in individuals experiencing psychological challenges, it is notable that these effects were observed in a community sample of adolescents, including eight participants (13.1%) with parent-reported ADHD, autism spectrum disorder, or both, reflecting the inclusion of neurodiverse individuals commonly encountered in community populations. Further, although females comprised the majority of participants (67% female, 25% male, and 7% nonbinary or gender-fluid), the inclusion of male and gender-diverse adolescents resulted in a more balanced gender distribution than is typical of many human–animal interaction studies^51^. Together with previous reports demonstrating benefits in individuals without known mental health conditions^6–8^ these findings suggest that brief human–animal interactions may support emotional regulation beyond therapeutic settings and serve as a low-intensity preventive resource in community populations.

Initial results established that the interaction in general produced measurable changes in emotional state, but they did not explain why the magnitude of those changes varied across participants. To address this, we experimentally manipulated human interaction style to isolate variation in human behavior and evaluate its effects within the context of the interaction as a whole. Accordingly, the design cannot isolate whether these effects arise specifically from animal behavior or the human’s tactile experience, nor can it disentangle how these components are perceived and integrated by the participant. Instead, participants’ emotional responses likely reflect their interpretation of the interaction, shaped by both the animal’s behavior and the human’s sensory experience. By preserving the integrated nature of the interaction, the findings provide evidence that variation in interaction dynamics is systematically associated with variation in emotional outcomes.

This approach builds on several established frameworks in the human-animal interaction literature. The Human-Animal Bond (HAB) framework emphasizes the human benefits that arise from positive interactions between humans and animals^38^, and the field of AAI has documented improvements in wellbeing across diverse populations^2–14,52,53^. At a broader level, integrative frameworks such as One Health and One Welfare emphasize that human, animal, and environmental wellbeing are interconnected^54,55^, highlighting the importance of cross-species interdependence. However, these frameworks are primarily articulated at conceptual or policy levels and place less emphasis on the behavioral processes unfolding within individual interactions^56^.

Complementary frameworks have begun to delineate potential mechanisms underlying these effects. The biophilia hypothesis and neurobiological models of tactile interaction suggest that humans are predisposed to respond positively to animals through attentional and physiological pathways^57,58^. The emotional contagion or emotional transfer hypothesis proposes that affective states may be shared across individuals and species^59–61^, emphasizing the reciprocal nature of AAIs. However, most studies focus primarily on human responses, and the animal’s experience is often viewed as an ethical prerequisite or experimental constraint^16^. A small number of studies have documented physiological coupling between humans and animals, including alignment in heart rate variability and hormonal responses during interaction^62–67^. Although these findings suggest that humans and animals may influence each other’s emotional states during AAIs, the potential for behavioral dynamics between humans and animals to affect human emotional outcomes has rarely been addressed.

The present findings address this gap by proposing a Two-Way Hypothesis, in which the animal’s behavior serves as an intermediate step linking human behavior to human emotional outcomes. By incorporating the animal as a responsive participant whose behavior varies as a function of human interaction, this approach reframes human-animal interactions as dynamic, reciprocal processes. It provides a tractable means of investigating how interaction dynamics between species shape psychological outcomes. In this framework, human interaction method functions as an upstream driver of those dynamics. Conceptually, it aligns with emerging perspectives in systems biology, interactive cognition, and social neurobiology that emphasize reciprocal processes across interacting systems^68–73^.

To test this idea experimentally, we manipulated tactile interaction style, thereby altering how participants engaged with animals, and assessed its effects on equine behavior. As predicted, scratching on the neck was associated with a higher observed frequency of affiliative lip-wiggling behavior (75.0% vs. 48.1%) and a substantially lower likelihood of voluntary disengagement (41.7% vs. 88.9%) than gentle petting on the back. These results are consistent with prior work demonstrating that horses exhibit differential responses to tactile stimulation depending on body region and contact style. Specifically, they respond positively to human touch that mimics common allogrooming behavior, such as firm contact on the neck^26^. In contrast to the effect observed for walking away, the difference in lip wiggling did not reach statistical significance in the clustered model. However, the estimated effect was in the predicted direction and associated with a large odds ratio, consistent with the broader pattern of effects across outcomes. Taken together, these results indicate that even modest, welfare-conscious differences in human behavior can alter animal responses within an interaction.

Having established that the manipulation altered equine behavior, we next tested whether these human-driven differences in equine responses were associated with variation in human outcomes. Adolescents who observed affiliative lip-wiggling behavior reported greater reductions in loneliness than those who did not, a pattern that aligns with the expectation that greater equine engagement would be associated with more pronounced positive emotional change. However, the relationship between equine disengagement and human emotional outcomes was more complex. Adolescents who observed an equine walk away from them during interaction, a behavior that might be expected to attenuate beneficial effects, instead reported greater reductions in sadness. It is important to note that sadness decreased across all participants regardless of equine disengagement, indicating that this pattern reflects differences in the magnitude of the benefit rather than its presence.

Several interpretations may account for this result, each pointing to how disengagement might shape the experience of the interaction rather than its overall value. Observing an animal exercise voluntary choice may be perceived positively, reinforcing a sense of authenticity or agency in the interaction^74–76^. Alternatively, brief interruptions in contact may alter the structure of the interaction itself, reducing anticipatory uncertainty or social evaluation concerns^47–50^. These mechanisms were not directly tested in the present study, but they suggest promising directions for future work to disentangle how specific features of interaction dynamics, including brief disengagement, shape the magnitude of emotional change.

Together, these findings support the Two-Way Hypothesis by suggesting that variability in outcomes across animal-assisted interventions may arise from differences in how humans engage with animals during interaction. By shaping animal responses, human behavior may contribute to downstream differences in emotional outcomes, providing a mechanistic account for the heterogeneity observed across studies.

These findings extend a growing literature on the beneficial role of equines in animal-assisted interventions. While horses have been widely incorporated and are relatively well studied^17–19^, donkeys and miniature equines have received comparatively less attention in controlled experimental research, despite their increasing use in practice^77–80^. Donkeys may offer unique advantages for AAIs due to their generally calm demeanor and lower reactivity in unfamiliar environments^81^. At the same time, their heightened tactile sensitivity suggests that the form of human contact may meaningfully influence their experience during interactions^81^. Consistent with this trait, some studies report positive mental health effects^78,82^, whereas others report stress-related behaviors during certain forms of human contact^28^.

To our knowledge, experimental studies examining miniature donkeys in intervention contexts are extremely limited, and only a small number of studies have evaluated miniature horses in this way^80^. The results of the present study demonstrate that miniature equines are capable of producing measurable emotional benefits even during very brief interactions. They possess a unique combination of physical and behavioral characteristics that may offer practical advantages, including reduced physical risk, greater feasibility for ground-based interaction formats, and lower resource requirements. At the same time, equines, including miniature horses and donkeys, exhibit substantial individual variation in behavior and responsiveness to humans, including strong preferences and occasional resistance to human-directed interaction, which should be considered in the design and interpretation of interventions^28,81,83–85^.

More broadly, these findings suggest that human benefits are not independent of the animal’s behavior, but arise through it. This relationship links animal welfare and human outcomes, such that conditions that support animal comfort and voluntary engagement may also enhance intervention effectiveness. Animal welfare therefore emerges not only as an ethical consideration, but as a mechanistic component of how human–animal interactions produce their effects.

Building on these findings, future work should consider several factors that will further refine how interaction processes are measured and tested. One consideration is sample size. Although the sample in the present study was substantial relative to much of the existing literature, it remained modest for some of the analytic questions addressed here (*n* = 61 / 51). Even so, effect sizes were sufficient to detect consistent patterns across analyses. Another consideration is broader characterization of the animals themselves. This study involved a small, consistent group of equines to enhance control over the interaction environment and reduce extraneous variability, but in doing so, limited assessment of species- or individual-level effects. Within this context, equine behavior was measured through participant report rather than independent coding. Participants received standardized training to support accurate reporting; however, these measures reflect perceived rather than directly observed behavior and may be influenced by participants’ own emotional states, attentional biases, or expectations. At the same time, participant-reported behavior captures an ecologically relevant dimension of the interaction, as the cues participants notice are reported to be most influential for their emotional responses^35–38^. Physiological measures also warrant further refinement. Heart rate did not show corresponding pre-post changes, suggesting that short-term emotional shifts may occur without detectable changes in heart rate in brief interaction contexts. This may reflect limited sensitivity of heart rate at this timescale for low-intensity interactions or the influence of movement-related noise during the interaction that obscures more subtle physiological effects. Finally, the study focused on short-term change following a single interaction, leaving longer-term and cumulative effects to be determined.

In summary, this study demonstrates that even brief interactions with miniature equines can improve adolescent emotional state, and that variation in equine engagement corresponds to variation in specific emotional outcomes. By experimentally linking human behavior to animal responses and, in turn, to differences in emotional change, these findings provide an empirical test of the Two-Way Hypothesis and support a model of human-animal interaction as a reciprocal process. Within this model, human behavior shapes animal responses, which are associated with downstream human outcomes. By explicitly positioning animal behavior as a measurable intermediate component of this process, the present work shifts the field beyond exposure-based accounts toward an interaction-based framework. This approach establishes a tractable foundation for a more mechanistic and predictive science of human-animal interaction.

## Supplementary Information

Below is the link to the electronic supplementary material.

Supplementary Material 1

Supplementary Material 2

## Data Availability

All datasets and analysis code used in this study are publicly available at: https://github.com/BeccaCalisi/equine-interaction-two-way-hypothesis.
