# Supplementary material for: Mutual wellbeing in an equine-assisted intervention: experimental evidence for the two-way hypothesis and the importance of interaction method
Source: Sci Rep. 2026 Sep 15;16:27658. doi: 10.1038/s41598-026-65168-2 (PMC13578761; doi:10.1038/s41598-026-65168-2)
Supplement: Supplementary file 1 [file 41598_2026_65168_MOESM1_ESM.pdf]

Date:____________ Time:____________ Group #:____ Code Name:____________ Survey (Post)

Please circle a number to indicate how you feel about the following statements.

1. I feel nervous or anxious

1	2	3	4	5	6	7
Not at all	A tiny bit	A little	Somewhat	Quite a bit	Strongly	Extremely

2. I feel sad

1	2	3	4	5	6	7
Not at all	A tiny bit	A little	Somewhat	Quite a bit	Strongly	Extremely

3. I feel excited

1	2	3	4	5	6	7
Not at all	A tiny bit	A little	Somewhat	Quite a bit	Strongly	Extremely

4. I feel lonely

1	2	3	4	5	6	7
Not at all	A tiny bit	A little	Somewhat	Quite a bit	Strongly	Extremely

5. I feel calm

1	2	3	4	5	6	7
Not at all	A tiny bit	A little	Somewhat	Quite a bit	Strongly	Extremely

After filling out the survey, please enter the values from the oximeter below:

Top number (blood oxygen saturation):________%SpO2

Bottom number (heart rate):________bpm

Please circle either "Yes" or "No" to answer the following questions about the animals' behavior:

1. Did you notice any of the following animals' lips wiggling while you interacted with them?

Mary (minidonkey):		Memphis (minidonkey):		Randy (minihorse):	
Yes	No	Yes	No	Yes	No

2. Did you notice any of the following animals walking away from you when you tried to touch them?

Mary (minidonkey):		Memphis (minidonkey):		Randy (minihorse):	
Yes	No	Yes	No	Yes	No
